# The development of a culturally sensitive educational video: How to facilitate informed decisions on cervical cancer screening among Turkish‐ and Moroccan‐Dutch women

**DOI:** 10.1111/hex.13545

**Published:** 2022-07-21

**Authors:** Nora Hamdiui, Martine P. A. Bouman, Mart L. Stein, Rik Crutzen, Damla Keskin, Amina Afrian, Jim E. van Steenbergen, Maria E. T. C. van den Muijsenbergh, Aura Timen

**Affiliations:** ^1^ National Coordination Centre for Communicable Disease Control Centre for Infectious Disease Control, National Institute for Public Health and the Environment Bilthoven The Netherlands; ^2^ Department of Primary and Community Care, Radboud University Medical Center Radboud Institute for Health Sciences Nijmegen The Netherlands; ^3^ Center for Media & Health Gouda The Netherlands; ^4^ Erasmus Research Centre for Media, Communication and Culture (ERMeCC) Erasmus University Rotterdam Rotterdam The Netherlands; ^5^ Department of Health Promotion, Care and Public Health Research Institute (CAPHRI) Maastricht University Maastricht The Netherlands; ^6^ Centre for Infectious Diseases Leiden University Medical Centre Leiden The Netherlands; ^7^ Pharos: Dutch Centre of Expertise on Health Disparities, Program Prevention and Care Utrecht The Netherlands; ^8^ Athena Institute for Research on Innovation and Communication in Health and Life Sciences VU University Amsterdam Amsterdam The Netherlands

**Keywords:** cervical cancer screening, entertainment–education, intervention development, The Netherlands, Turkish‐ and Moroccan‐Dutch women, video production

## Abstract

**Background:**

In the Netherlands, all women aged 30–60 years are invited to participate in the national cervical cancer screening programme, which is aimed at early detection and treatment of precancerous lesions. One fourth of the Dutch population has a migration background, with Turkish and Moroccan immigrants being the largest immigrant populations. Turkish‐ and Moroccan‐Dutch women show lower screening participation rates and a higher incidence of cervical cancer, compared to native Dutch women. Since current information materials are not tailored to these women's needs, we developed a short culturally sensitive educational video to facilitate informed decision‐making for cervical cancer screening among Turkish‐ and Moroccan‐Dutch women. This article describes the development process of this video and the lessons learned.

**Methods:**

Using the Entertainment–Education communication strategy, we collaborated with an interdisciplinary team of Turkish‐ and Moroccan‐Dutch women, researchers, public health experts, and creative media professionals. We developed the video following the different stages of the Media Mapping model: Orientation, Crystallization, Design/Production, Implementation, and Dissemination. Each stage is described in the paper.

**Results:**

The video was developed in Moroccan‐Arabic, ‐Berber and Turkish, and emphasized three main themes: (1) more certainty about having cervical (pre)cancer and the possibility to prevent treatment, surgery, or premature death, and because of this, being there for the children, (2) according to the Islam, a woman should take good care of her health, and (3) anxiety, shame, and privacy.

**Conclusions:**

A short culturally sensitive educational video, delivered as part of a larger intervention together with the current information brochure, was developed based on theory and grounded in the needs of Turkish‐ and Moroccan‐Dutch women. The value and effectiveness of this intervention to facilitate informed cervical cancer screening decisions are evaluated in a randomised controlled trial.

**Patient or Public Contribution:**

We collaborated with Turkish‐ and Moroccan‐Dutch women during the development process of a short culturally sensitive educational video. Turkish‐ and Moroccan‐Dutch women were also invited to watch the raw footage to verify whether the content and presentation matched their needs and requirements.

## 1 INTRODUCTION

Since 1996, a national cervical cancer (CC) screening programme has been implemented in the Netherlands. Although CC screening has led to a substantial decline in both early‐ and late‐stage CC,^1^ Turkish‐ and Moroccan‐Dutch women show lower screening participation rates compared to native Dutch women.^2^

Based on the national screening programme, regional screening organizations invite women aged 30–60 years every 5 years by sending them a Dutch invitation letter and information brochure to their home addresses. This information brochure is also available in other languages (i.e., English, Arabic, and Turkish), but can only be found online (via the URL provided in the Dutch invitation letter). Other additional information materials (e.g., infographics, factsheets, and videos) can also be found online.

We found earlier that the current invitation letter and information brochure are not tailored to the needs of Turkish‐ and Moroccan‐Dutch women because of a lack of information on the practical, emotional, cultural, and religious aspects of CC screening.^3^ According to the Elaboration Likelihood Model (ELM), depending on their motivation and skills, individuals engage in high or low elaboration (i.e., level of effort to process) when encountering a message.^4^ Watching a short video is a relatively small effort compared to reading an invitation letter and information brochure. More importantly, a video may ‘prime’ an individual to search for and read additional information, or spark conversations about the topic with others. To date, no such video is in place. Therefore, we decided to develop a short culturally sensitive educational video to facilitate informed decision‐making regarding CC screening participation. In this article, we describe the development process of this video and the lessons learned.

**Figure 1:**
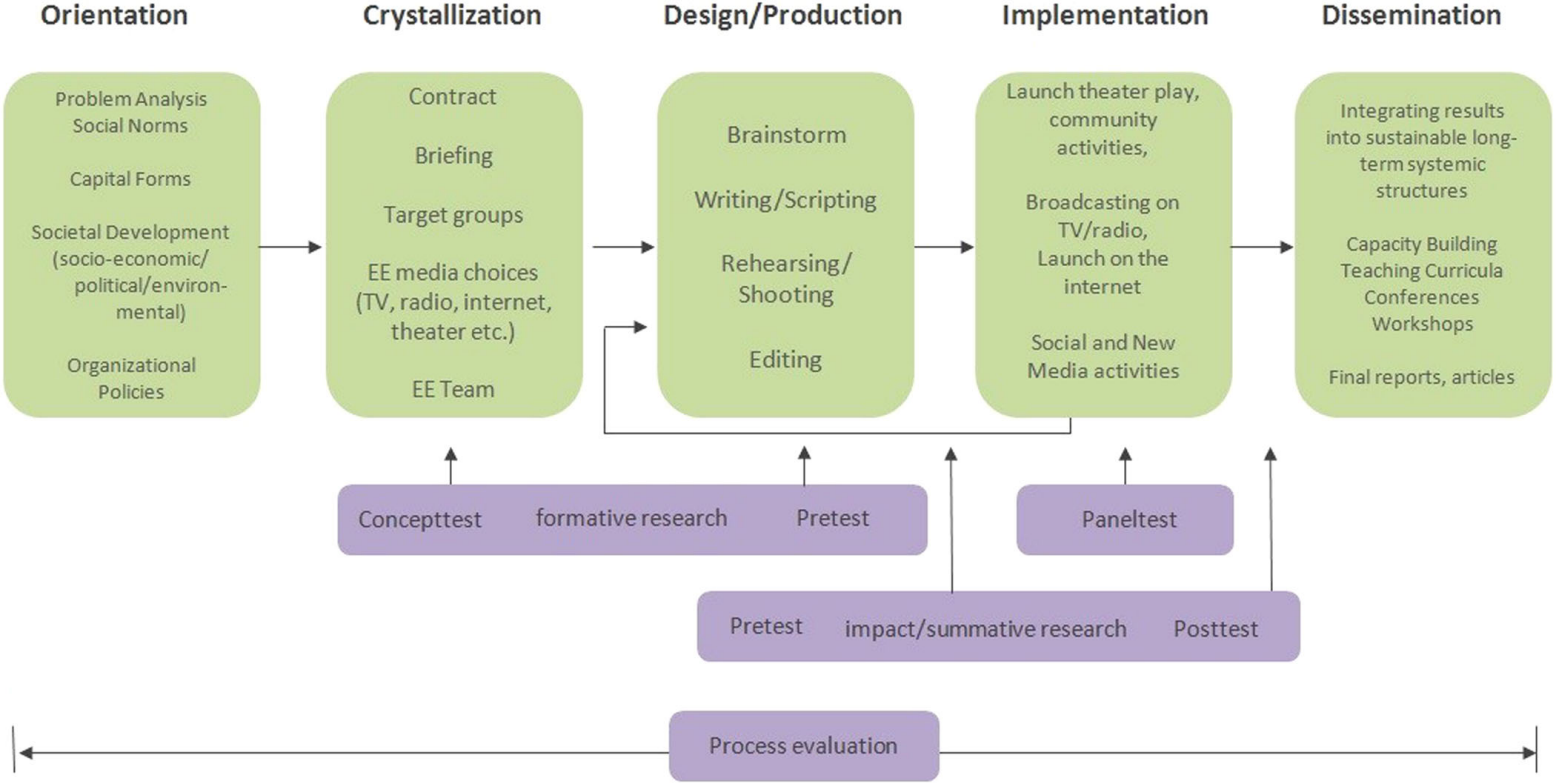
Media Mapping model. Copyrights: Center for Media & Health, adapted from Bouman, 1999.^6^

To create the video, an interdisciplinary team was formed consisting of members of the targeted audiences (i.e., Turkish‐ and Moroccan‐Dutch women between 30 and 60 years old), researchers, public health experts, and creative media professionals. We will illustrate this collaborative process following the different stages of the Media Mapping model of the Center for Media and Health^5^: Orientation, Crystallization, Design/Production, Implementation, and Dissemination (see Figure 1). Parts of this Media Mapping model are formative and summative research.

## 2 MATERIALS  AND METHODS

### 2.1 Orientation

The current strategy in the Netherlands is to encourage women to gather information about both the advantages and disadvantages of CC screening, to ultimately make an informed decision regarding their participation. Participation in CC screening is entirely voluntary.

In the Netherlands, 24.4% of the population has a migration background, which includes both individuals who were born abroad (further referred to as first‐generation immigrants) and those who have at least one parent born abroad (further referred to as second‐generation immigrants).^7^, ^8^, ^9^ Overall, Turkish and Moroccan immigrants are the largest immigrant populations, with 422,030 persons and 414,186 persons, respectively, each accounting for 2.4% of the total population in 2021 in the Netherlands.^10^

Steens et al.^2^ estimated that in the Netherlands, 64% of women born in Turkey and 53% of women born in Morocco participated in CC screening, compared to 79% of women born in the Netherlands. These lower CC screening participation rates are worrisome, especially because these populations also show higher CC incidence ratios of 1.2 (Turkish) and 1.7 (Moroccan) compared to native Dutch women (with a reference incidence ratio of 1.0).^11^

Multiple factors can play a role in women's decision‐making process regarding cancer screening, such as lack of awareness and knowledge, organizational issues, and sociocultural aspects.^12^ According to the concept of informed decision‐making based on the Rational Decision Model, screening decisions are based on making optimal use of information and rationally weighing all aspects involved, considering both the pros and the cons.^13^, ^14^, ^15^ This means that uncertain benefits (e.g., longer duration of life if a precursor of cancer is successfully detected and treated) and risks of adverse effects (e.g., false‐positive and ‐negative test results, overdiagnosis and ‐treatment, and discomfort or pain) should be considered in the individual's decision‐making process.^13^, ^14^, ^15^ In a previous study using focus group discussions and a follow‐up questionnaire among Turkish‐ and Moroccan‐Dutch women, we found that they do not only consider factual medical information, but also practical, emotional, cultural, and religious aspects of CC screening before deciding to screen or not.^3^ Next to medical information (e.g., risk factors of CC), practical, emotional, cultural, and religious aspects play a significant role in the decision‐making process of these women. This is why we decided to combine an informative approach with a more affective approach.

Since the current invitation letter and information brochure include mostly factual medical information, practical, emotional, cultural, and religious aspects need to be added. Moreover, Turkish‐ and Moroccan‐Dutch women often indicated that they did not (thoroughly) read the invitation letter and information brochure, or simply could not understand these materials due to a lack of a good command of the Dutch language.^3^ Moreover, Turkish‐ and Moroccan‐Dutch make less use of printed media and more use of audio‐visual media (30% vs. 75%, and 34% vs. 72%, respectively).^16^ A previous study showed that culturally competent educational films improved the informed decision‐making regarding prenatal screening among Dutch multicultural pregnant women.^17^

Therefore, in accordance with the ELM, and to further stimulate the informed decision‐making for CC screening among Turkish‐ and Moroccan‐Dutch women, we developed a short culturally sensitive educational video in the women's own languages (Turkish, Moroccan‐Arabic and ‐Berber) to complement the currently used information brochure. The narrative in this video focused on practical, emotional, cultural, and religious aspects of CC screening.

#### 2.1.1 Theoretical framework

To develop a short culturally sensitive educational video to facilitate informed decision‐making, we made use of the Entertainment–Education (EE) communication strategy.^6^, ^18^ This strategy can be defined as ‘the process of purposively designing and implementing a mediating communication form with the potential of both entertaining and educating people, to enhance and facilitate different stages of behaviour change’.^6^ This strategy allows for a better alignment with the lifestyles and culture of audiences who lack a ‘reading culture’. Since Turkish‐ and Moroccan‐Dutch make less use of printed media and more use of audio‐visual media,^16^ we chose to create a video based on the EE strategy. The EE strategy is characterised by a more affective approach and applies storytelling to present (new) knowledge, ideas, norms, and practices with the aim of stimulating conversations with family, friends, and acquaintances about the issues raised in the video.^6^, ^18^, ^19^, ^20^ Based on the Social Cognitive Theory,^19^, ^21^ individuals may learn by observing the behaviour of role models seen in real life or by characters seen in films, which contribute to storylines effectively conveying specific knowledge, ideas, norms, and practices.^20^ To facilitate recognisability for all women watching the video, we chose to portray three types of role models in the video: one who has a positive attitude towards CC screening participation; one who has a negative attitude towards CC screening participation; and one who has an ambivalent attitude towards CC screening participation.

### 2.2 Crystallization

Upon completion of the orientation phase, we approached several video production companies. We selected the company called Zouka Media, who had proven experience and expertise in producing videos for and by Turkish‐ and Moroccan‐Dutch women. The ideas of the researchers and Zouka Media aligned well in approaching and informing these women through a short culturally sensitive educational video (e.g., type and content of the storyline, recruitment and type of actresses, and the setting of the video), and Zouka Media showed great willingness to work in an interdisciplinary team. Subsequently, a contract was negotiated between the partners.

We conducted formative research as input for the development of the briefing document. We held six focus group discussions among 24 Turkish‐ and 20 Moroccan‐Dutch women, during which seven main themes emerged.^3^ These themes were (1) (informed) decision‐making and information need, (2) religious beliefs and values, (3) cultural beliefs and values, (4) perceived threat of disease and fear of cancer and death, (5) subjective norm and social support, (6) practical factors, and (7) self‐sampling. To identify the most relevant themes to include in our video (as including all seven themes would result in a too lengthy video), we developed a follow‐up questionnaire and distributed this via offline and online snowball sampling. In total, this questionnaire was completed by 248 Turkish‐ and 234 Moroccan‐Dutch women. During analyses, we used the Confidence‐Interval Based Estimation of Relevance approach to select the most relevant determinants of informed decision‐making and inform the focus of the video.^22^ As a result, we identified four relevant determinants, that is, beliefs about (1) preventing treatment or surgery, (2) reducing the chance of dying from CC, (3) shame regarding the cervical smear test performed by the general practitioner, and (4) privacy regarding the smear test. Based on the findings of this formative research, we developed a briefing document in close collaboration with the video producer. This document contained information on the background, target groups, objectives, planning, organisation, format, themes, and final delivery.

Thereafter, an EE team was formed, consisting of representatives of the targeted audiences, the health communication field, and the media field. In the end, the team consisted of eight Turkish‐ and Moroccan‐Dutch women (who later figured as the main characters in the videos), a health communication expert, three cancer screening and public health experts (with Turkish and Moroccan cultural and linguistic backgrounds), and a video producer and director (both with a Moroccan cultural and linguistic background).

We developed the video in three languages, namely, Turkish, Moroccan‐Arabic, and ‐Berber (all with Dutch subtitles). Depending on the age, educational level, and migration generation, Moroccans in the Netherlands generally speak Dutch, Moroccan‐Arabic, and/or ‐Berber. Since Berber languages are for them solely speaking languages, Moroccan‐Berber women with a limited command of the Dutch language will have difficulties with reading the Dutch information brochure. They will, however, be able to watch the Moroccan‐Berber video. The same holds for Moroccan women who speak solely Moroccan‐Arabic and Turkish women; they are able to watch the Moroccan‐Arabic and Turkish video, respectively.

## 3 RESULTS

### 3.1 Design/Production

We identified four relevant determinants and decided to choose for three main themes, since shame and privacy (i.e., determinants 3 and 4) could easily be discussed in the video jointly. As a result, the short video emphasized the following themes: (1) ‘more certainty about having cervical (pre)cancer and the possibility to prevent treatment, surgery, or premature death, and because of this, being there for the children’, (2) ‘according to the Islam, a woman should take good care of her health’, and (3) ‘anxiety, shame, and privacy’. To facilitate informed decision‐making, it was important to ensure balanced content in terms of the potential benefits and adverse effects of CC screening. We also wanted to emphasise the ease and reliability of self‐sampling, as since 2017, women are able to participate in CC screening by collecting a sample by themselves (i.e., self‐sampling). As many women indicated not having heard of self‐sampling before,^3^ the possibility of self‐sampling was introduced in the video.

Thereafter, we incorporated two main themes regarding self‐sampling. The first theme was ‘it is easy and not painful to perform self‐sampling’. This was incorporated into the script: ‘I also did it [self‐sampling] at home. It was so easy. It was done in a heartbeat’. The second theme was ‘trust in oneself to correctly perform self‐sampling and trust in the test result’. This was illustrated in the script by one of the actresses saying: ‘Did you know that you can also request a self‐sampling kit? Then you can do it at home without someone else being around. The doctor told me that it is just as reliable as the one performed by the doctor’. In Table S1, examples of quotes from the video can be found for each theme.

Because of the respective age range of 30–60 years, we decided to cast three semi‐professional actresses aged 30, 40, and 50 years approximately. We chose to work with semi‐professional actresses to facilitate a natural look and feel of the video, and also due to our time frame and budget. The youngest actress (aged approximately 30 years) would be the doubting character: the one who was not sure what to decide, since she received the invite to participate in CC screening for the first time. The oldest actress (aged approximately 50 years) would represent the positive role model; she is the most experienced one, as she had already participated in CC screening a couple of times. The actress aged approximately 40 years would represent the negative role model.

Since nonparticipation can also be an outcome of an informed decision, we decided that the video would end with the character in doubt saying that she will search for more information to make her decision. The goal of the short video is to stimulate women to make an informed decision by searching for information or talking about the subject with others or the general practitioner. The pay‐off, therefore, included ‘Get well informed. Talk about it with a friend or call your general practitioner. Would you like to know more? Go to…’.

We instructed the actresses to act as close friends with whom you can discuss this (intimate) subject, since we know that close friends can act as emotional support in stimulating someone to take the last steps in going to the general practitioner when the CC screening procedure is regarded as scary.^3^ Close friends can also provide verbal support when women lack a good command of the Dutch language and are not able to make an appointment.^3^ In addition, we chose to film the video in a hair salon of one of the friends after closing time, as the EE team thought that these kinds of subjects may then be discussed (in a safe environment) with only women. It is also possible to talk about other subjects (e.g., hair) in between to make it entertaining, less formal, and educational.

After having multiple brainstorming sessions with the EE team and the research team to share professional knowledge as input for the video script, we started the production. Because of the different languages, we worked with three different main characters per video. One of the main characters was a Moroccan woman who spoke both Moroccan‐Arabic and ‐Berber and played a main character in both the Moroccan‐Arabic and ‐Berber versions of the video. Each team of actresses had two sessions before the shooting day with a health communication professional and two creative media professionals to become familiar with the Dutch script. During the first session, we discussed the goal of the video, how they were expected to interact with each other, how to translate some words used in the script, and what they were expected to wear during the shooting day (i.e., no or neutral make‐up and neutral and matching colours of their clothing). At the end of this first session, we asked the three teams of actresses to practise and rehearse with each other and play out the script during the second session. During this second session, the two Moroccan teams performed the storyline very well and were given only small additional comments for the shooting day. Unfortunately, the Turkish team had insufficient preparation time due to illness in the team and scheduling difficulties and proved to be not well adjusted to each other during the performance. They were, therefore, asked to meet (virtually) again to become familiar with the script and practice the lines and interactions with each other. To help the team do this, one of the creative media professionals offered to be present during this practice session for guidance and support.

**Figure 2:**
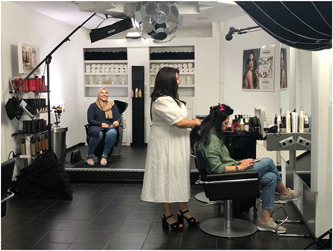
Shooting day 1: Moroccan‐Arabic group.

**Figure 3:**
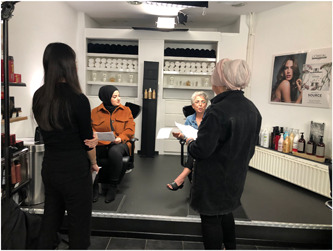
Shooting day 2: Turkish group.

Unfortunately, because of lockdown measures to control the COVID‐19 pandemic, the initially planned shooting days (March 2020) were cancelled. After these measures were lifted, we held two days of shooting under strict restrictions in June 2020 (see Figures 2 and 3). These restrictions included a maximum of 10 persons at the filming location, as few movements of the actresses as possible, social distancing (i.e., maintaining a distance of 1.5 m) between all persons at the filming location (the hairdresser and the client were exempted, since contact‐based professions were allowed again), and, although not nationally compulsory, face masks covering the nose and mouth had to be worn by all persons at the filming location (actresses were exempted). Additionally, general hygiene measures had to be taken into account (e.g., regularly and thoroughly washing hands, avoiding touching eyes, nose, and mouth, covering the mouth and nose with the bent elbow when coughing or sneezing, and cleaning and disinfecting surfaces frequently).

After the production and before the final editing, we invited a convenience sample of five Turkish‐ and Moroccan‐Dutch women to watch the raw footage to verify whether the content and presentation matched their needs and requirements. Meetings were also organised with various experts on CC, health communication, public health, and Turkish and Moroccan languages/culture (e.g., gynaecologist, general practitioner, psychologist, cancer screening programme expert, health communication experts, public health experts, and key community informants) to provide feedback. We asked them for detailed comments and suggestions on its content and presentation, and to assess its understandability and cultural appropriateness. Although their feedback was overall positive, the following changes were suggested: (1) to add culturally appropriate background music, (2) to add certain shots, delete redundant information, shorten the video (e.g., the first shot of the hair salon), or replace certain shots (e.g., when one of the jokes did not work out), (3) to speed up the Turkish conversation in the editing, and (4) to overlay audio records in the pay‐off of the video.

After three editing rounds, three short videos (of 3–4 min) were delivered in July 2020. Each can be found through https://youtu.be/e8m3vz7OHz0 (Turkish), https://youtu.be/QMWwWc4gS4o (Moroccan‐Arabic), and https://youtu.be/VP8Gl2Na4nI (Moroccan‐Berber). An overall making‐of video (in Dutch) can be found via https://youtu.be/uwq1yhRmKV4.

### 3.2 Implementation

We studied the video's effectiveness on the women's informed decision‐making through a two‐arm randomised controlled trial (RCT) (see the Netherlands Trial Register, nr NL8453). In this trial, respondents either received the current Dutch information brochure (i.e., normally sent to their home addresses every 5 years and can be considered ‘usual care’) (control condition), or this brochure combined with our short video (intervention condition). We were particularly interested in finding out whether a combination of an information brochure (informative approach) and a video (affective approach) would better support the informed decision‐making regarding CC screening participation among Turkish‐ and Moroccan‐Dutch women. The manuscript including the results of this trial is currently in press.^23^

Initial respondents were recruited via several social media platforms and invited to complete an online questionnaire. Following respondent‐driven sampling, respondents were asked to recruit a number of peers from their social network to complete the same questionnaire. Respondents were asked several questions on sociodemographic characteristics and informed decision‐making. Subsequently, respondents were randomised to one of the two conditions (i.e., arms). Respondents in both the intervention and control arms were asked to study the brochure that was visualised using an image per page. The 20 images could be studied through a ‘slider’ on our questionnaire webpage. Moroccan women in the intervention arm could thereafter indicate which language they preferred: Moroccan‐Arabic or ‐Berber. By clicking ‘Next’, a YouTube video in the preferred language could be played (and if preferred, a full‐screen viewing modus could be chosen). For Turkish women, a YouTube video in Turkish was presented. After completing one of the two conditions, questions on informed decision‐making were asked again. The final sample of this trial, focusing on the effectiveness of the video on informed decision‐making, included 686 Turkish‐ and 878 Moroccan‐Dutch women. Of this sample, 793 were randomised to the control group (350 Turkish and 443 Moroccan) and 771 to the intervention group (336 Turkish and 435 Moroccan).

Respondents in the intervention arm were also asked what they thought about the video.

Overall, most women (both Turkish and Moroccan) scored the video as entertaining, informative, clear, and good.

In the Turkish video, the older actress was portrayed without a headscarf (she did not wear this in real life) and she was coincidentally the positive role model with the most CC screening experience and knowledge. Less than five Turkish respondents found the video offensive to women wearing headscarves, of whom one of them said: ‘The framing where the woman without a headscarf explains everything to the young ignorant women with headscarves is biased and derogatory’.

The distribution of the role models was solely based on the actresses' age and we did not take their physical appearance (neither in real life nor in the video) into account. The acting skills in the Turkish video also had a lower quality than in the Moroccan video. Whether and how these characteristics may affect the video's effectiveness on informed decision‐making are evaluated in our RCT.

### 3.3 Dissemination

After the end of the trial, the three short culturally sensitive educational videos were made publicly available on the website of the National Institute for Public Health and the Environment.^24^ Currently, the invitation letter that is sent to women aged 30–60 years includes a hyperlink that refers to all additional information materials including these videos.

The videos have also been distributed via the Dutch expertise centre called Pharos. Pharos has ample experience with adapting and disseminating health‐promoting materials among immigrants in the Netherlands. The videos can be found on the Pharos website^25^ and the website for general practitioners that Pharos hosts together with the Dutch college of general practitioners.^26^

## 4 DISCUSSION

In this article, we describe the development of a short culturally sensitive educational video for Turkish and Moroccan women in the Netherlands. This video (affective approach), together with the current Dutch information brochure (informative approach), aims to facilitate informed CC screening decisions among Turkish‐ and Moroccan‐Dutch women. In agreement with earlier calls for full disclosure of intervention development and content,^27^ this article provides an overview of the development process and the content that was produced. Other similar video productions and studies may learn from our systematic approach and our lessons learned when developing health‐related videos for immigrant populations.

Our participatory EE strategy using the Media Mapping model provides a framework that is both grounded in theory and practice, and involved both members of the intended audiences and various experts. Both the EE strategy and the Media Mapping model proved to be valuable tools to guide the development of a short culturally sensitive educational video.

We, therefore, recommend designing and developing such an intervention in a systematic manner (using e.g., the Media Mapping model) with an interdisciplinary team of members of the targeted audiences, researchers, public health experts, and creative media professionals to ensure the quality and cultural appropriateness of such an intervention.

During this intervention development process, we have learned a few important lessons.

First, although Turkish‐ and Moroccan‐Dutch women were closely involved in the development process, comments were made by less than five Turkish respondents about specific role models wearing a headscarf or not. As described earlier, in the Turkish video, the older actress was a woman who was portrayed without a headscarf and she was the positive role model with the most CC screening experience and knowledge. We based the distribution of the role models solely on the actresses' age. We used age as an indicator for the number of times invited for screening, and thus, the number of times that a decision had to be made. The more times invited, the more a woman may have read, talked, or thought about or have experiences with the screening. In the future, in addition to age, one's physical appearance (e.g., wearing a headscarf or not) should be thoroughly considered when assigning role models to minimise stereotyping as much as possible. It is important to note, however, that it is very challenging to develop a single video tailored to the needs of a diverse range of individuals (e.g., young/old, first/second generation, with a low/high educational level). Having said this, efforts should be made to ensure content and image appropriateness for the majority of the targeted audience.

Second, and in line with the previous lesson, due to costs, we could only develop a single video (in three different languages), while a broad age range of 30–60 years might require multiple videos, as women in this age group differ greatly in sociodemographic characteristics, such as Dutch language proficiency, educational level, and migration generation. During our previous studies in this project (on which we based the content of the video), we included mostly first‐generation immigrant women aged 46–60 years with a low or medium educational level who came together at community venues.^3^ As a result, the themes in the video may be more directed to these women and may not address other Turkish‐ and Moroccan‐Dutch women between 30 and 60 years old. In line with this, some Moroccan respondents mentioned that the video seems to be directed at those not able to understand the Dutch language. We expect that our RCT can reveal whether or not the effect of the video differs for several subgroups.

Third, because of our affective approach, the idea was to portray the characters in the video as close friends who talk about intimate matters and give each other advice and emotional support. Because of the COVID‐19‐related restrictions during the shooting days (keeping 1.5 m distance), the three actresses were, however, not able to act as close friends. The actresses could not sit close to each other and could not touch, hug, or kiss each other. We think that this explains why some of the respondents could not imagine that such a conversation would take place in real life. The actresses might have looked like three random strangers, which makes it indeed unlikely that they would discuss such an (intimate) subject with each other.

Fourth, the acting skills in the Turkish video had a lower quality than in the Moroccan video caused by insufficient preparation time to feel comfortable acting with each other, as we already observed during our second session with the actresses before the shooting day. Although we kindly asked the Turkish actresses after the second session to put in more time and effort to get a smooth storyline on the shooting day, it remained difficult for them to remember the lines and act naturally. A great advantage in the Moroccan teams of actresses was that they already knew each other (and some were even actual close friends). We, therefore, advise allowing for sufficient time to practise acting with each other, especially if actresses do not already know each other.

Furthermore, differences in work culture, expectations, and perspectives between representatives of the health communication field and the media field can result in misunderstandings and challenges in combining and balancing entertainment and education. To avoid misunderstandings, we invited all team members to articulate their ideas and views and explain their importance and relevance for the final product. As both fields (i.e., health communication and media) are responsible for a balanced mixture of entertainment and education, we made agreements on the roles and responsibilities of both fields, in each stage of the development process. We invested time to create mutual understanding and to reach consensus about the final product's form, content, and presentation.

Finally, the approved original script may change due to technical or practical production matters (e.g., COVID‐19‐related restrictions), casting, and costs. As an example, due to the restrictions on the filming location, we were not able to visualise a safe nor homely environment in which close friends discuss intimate subjects, such as cancer and the smear test performed by the general practitioner. Time to prepare the shooting days was also limited, since we had to wait for COVID‐19‐related measures to be lifted and quickly plan the shooting days due to the upcoming summer break and other upcoming film projects of the video producer. Such adjustments need to be discussed, agreed upon, and made during the ongoing production process, keeping in mind that, in culturally sensitive educational videos, alterations might have a stronger effect on the impact, than in written factual information provision.

## 5 CONCLUSION

In the Netherlands, women aged 30–60 years are invited to participate in a free‐of‐charge national CC screening programme, but Turkish‐ and Moroccan‐Dutch women show lower screening participation rates and a higher incidence of CC, compared to native Dutch women. The current information materials are not tailored to these women's needs, since there is a lack of information on the practical, emotional, cultural, and religious aspects of CC screening.^3^ We, therefore, developed a short culturally sensitive educational video to facilitate informed decision‐making for CC screening among Turkish‐ and Moroccan‐Dutch women. The value and effectiveness of this video, delivered as part of a larger intervention together with the current information brochure, are evaluated in an RCT.

## AUTHOR CONTRIBUTIONS

*Conceptualised and supervised the project*: Nora Hamdiui, Mart L. Stein, Jim E. van Steenbergen, Maria E. T. C. van den Muijsenbergh, Aura Timen. *Developed and tested the short culturally sensitive educational video*: Nora Hamdiui, Martine P. A. Bouman, Damla Keskin, Amina Afrian. *Wrote the first version of the manuscript*: Nora Hamdiui. *Critically reviewed and substantively revised the manuscript multiple times*: Nora Hamdiui, Martine P. A. Bouman, Mart L. Stein, Rik Crutzen, Damla Keskin, Amina Afrian, Jim E. van Steenbergen, Maria E. T. C. van den Muijsenbergh, Aura Timen. All authors read and approved the final manuscript.

## CONFLICTS OF INTEREST

None

## Supporting information

Supporting information.

## Data Availability

Data sharing is not applicable to this article, as no new data were created or analysed in this study.
