# Supplementary material for: The development of a culturally sensitive educational video: How to facilitate informed decisions on cervical cancer screening among Turkish‐ and Moroccan‐Dutch women
Source: Health Expect. 2022 Jul 21;25(5):2377–85. doi: 10.1111/hex.13545 (PMC9615061; doi:10.1111/hex.13545)
Supplement: Supplementary file 1 — Supporting information. [file HEX-25--s001.docx]

**SUPPLEMENTARY FILE**

*The development of a culturally sensitive educational video: how to facilitate informed decisions on cervical cancer screening among Turkish- and Moroccan-Dutch women*

**Table S1. Corresponding quotes per theme, included in our developed short video**

| **Theme** | **Examples of corresponding quotes in the video** |
| --- | --- |
| More certainty about having cervical (pre)cancer and the possibility to prevent treatment, surgery, or premature death, and because of this, being there for the children | *“I think it’s wise to participate in screening. Prevention is better than cure, right? It’s a small effort and you can save yourself a lot of misery.”* |
|  | *“Well, what do you prefer? Finding out that you are sick to get treated? Or to eventually (Allah may forbid) leave Salma [her daughter] all by herself?”* |
| According to the Islam, a woman should take good care of her health | *“Everybody should know for themselves, but Allah does urge you to take good care of your health.”* |
| Anxiety, shame, and privacy | *“Well, I am not spreading my legs for a man, not even for a doctor. That’s so embarrassing. I have also heard that it [sampling procedure] hurts.”* |
| It is easy and not painful to perform self-sampling | *“I also did it [self-sampling] at home. It was so easy. It was done in a heartbeat.”* |
| Trust in oneself to correctly perform self-sampling and trust in the test result | *“Did you know that you can also request a self-sampling kit? Then you can do it at home without someone else being around. The doctor told me that it’s just as reliable as the one performed by the doctor.”* |
